# Do COVID-19 Antibodies Provide Long-Term Protection?

**DOI:** 10.7759/cureus.12441

**Published:** 2021-01-03

**Authors:** Sheeba Ansari, Mubeen Memon, Ratan Kumar, Sidra Memon, Muhammad Khizar Memon

**Affiliations:** 1 Internal Medicine, Liaquat University of Medical and Health Sciences, Jamshoro, PAK; 2 Pulmonology, Liaquat University of Medical and Health Sciences, Jamshoro, PAK; 3 Internal Medicine, Civil Hospital Khairpur, Khairpur, PAK; 4 Internal Medicine, Jinnah Sindh Medical University, Karachi, PAK

**Keywords:** coronavirus, antibodies, pandemic

## Abstract

Introduction: Post severe acute respiratory syndrome coronavirus 2 (SARS-CoV-2) infection, an immune response is generated among healthy, immunocompetent individuals with immunoglobulins (IgG and IgM) antibodies. IgM rises earlier than IgG, indicating a recent infection. However, a detailed analysis is required to assess long-term immune reactions induced by antibodies.

Method and Materials: The study was conducted at a tertiary care hospital in Pakistan from June 2020 to October 2020 where serum samples were collected from patients. The samples were obtained by phlebotomy for antibody testing. All the reactive patients were followed up after 60 days of initial testing.

Results: A total of 728 patients participated in the study, of which 79​ (10.8%) were seropositive at baseline. Seventy-two (91.1%) participants came back for follow-up after 60 days (two months) and were included in the final analysis. Among the 72 participants, 35 (48.6%) exhibited symptoms of coronavirus disease 2019 (COVID-19) infection and 37 (51.4%) were asymptomatic. After 60 days, 37 (including 20 symptomatic and 17 asymptomatic) participants were still seropositive for SARS-CoV-2 enzyme-linked immunosorbent assay (ELISA) test. Mean change in percentage from seropositive to seronegative was more in asymptomatic compared to symptomatic patients (54.0% vs. 42.8%).

Conclusion: In this study, humoral immunity against SARS-CoV-2 is not long-lasting among individuals with mild signs and symptoms. Care should be taken while implicating that antibodies can provide long term protection against SARS-CoV-2. Further large-scale studies are needed.

## Introduction

Nearly all immune-competent individuals will develop an immune response following severe acute respiratory syndrome coronavirus-2 (SARS-CoV-2) infection. Like infections with other pathogens, SARS-CoV-2 infection elicits development of IgM and IgG antibodies, which are the most useful for assessing antibody response [1]. An assay that tests for IgM antibodies may detect a more recent infection with SARS-CoV-2, but typically both immunoglobulins (IgM and IgG) rise early in SARS-CoV-2 infections. IgM levels do wane earlier than IgG, and thus assays that test IgM alone may not detect prior infection [2]. Data ​generated through serological studies can greatly aid in supplementing the results from qRT-PCR (real-time quantitative reverse transcription polymerase chain reaction), as well as contribute to sero-epidemiology, which has been shown to help in the design of virus elimination programs [3].

It is not yet known if antibodies serve as an indication of the presence or absence of protective or sustained immunity. Negative SARS-CoV-2 antibody results do not rule out SARS-CoV-2 infection, particularly in those who have been in contact with the virus. Therefore, results from antibody testing should not be used as the sole basis to diagnose or exclude SARS-CoV-2 infection or to inform infection status [4]. In a recent study, the presence of anti-SARS-CoV-2 antibodies neither indicated the rapid eradication of the virus nor provided immune protection from disease deterioration [5]. Moreover, a few recovered patients that were discharged from hospitals tested positive in nucleic acid tests, it is still necessary to develop new sensitive and specific detection methods for the confirmation of virus-infected persons, carriers, and recovered patients [6].

In an ongoing pandemic with so much uncertainty about the virus and disease itself, there are many more discoveries yet to be known. Further studies on coronavirus disease 2019 (COVID-19) antibodies or serology need to support whether they are protective in long-term or not.

## Materials and methods

This longitudinal study was conducted at the outpatient department (OPD) of the Internal Medicine department at a tertiary care hospital in Pakistan from June 2020 to October 2020. Participants were enrolled from June 2020 until August 2020 via convenient consecutive sampling. Informed consent was also taken. Liaquat University of Medical and Health Sciences issued approval LUMHS/2020/R-CV-IRB-04.

Serum samples were collected from patients for anti-SARS-CoV-2 antibodies. The samples were tested using ELISA against the SARS-CoV-2 spike protein’s prefusion-stabilized extracellular domain [7]. A signal to threshold ratio of around 1:100 serum dilution with background correction of greater than 1.0 was considered reactive, however, higher antibody titers were demonstrated with higher ratios. This demonstrated that the assay was 99.8% specific and 100% sensitive [8].

Those who were seropositive were asked to come back after 60 days for follow up in September and October 2020. They completed a survey for symptoms of viral illness (fever, shortness of breath, cough, sore throat, fatigue, and loss of smell) experienced in previous two months. Phlebotomy for serology was repeated. 

We evaluated the change in the participants from seropositive to seronegative in 60 days. Data was analyzed using Statistical Package for Social Sciences (SPSS) version 23 (IBM Corp., Armonk, NY, USA).

## Results

After informed consent, 728 participants were enrolled in the study. Mean age of participants was 42 ± 17 years. There were 421 (57.8%) males and 307 (42.2%) females. Seventy-nine (10.8%) participants were seropositive at baseline. Seventy-two (91.1%) participants returned for follow-up after 60 days (two months) and were included in the final analysis. Among 72 participants, 35 (48.6%) were symptomatic and 37 (51.4%) were asymptomatic. After 60 days, 37 (51.3%) participants were seropositive for the SARS-CoV-2 ELISA test. Among symptomatic participants, out of 35 participants, 20 (54.1%) participants remained seropositive. Among asymptomatic participants, out of 37 participants, 17 (45.9%) participants remained seropositive. Mean change in percentage from seropositive to seronegative was more in asymptomatic compared to symptomatic patients (54.0% vs. 42.8%) (Table 1).

**Table 1:** Comparison of sero-positivity at baseline and at day 60 ELISA: enzyme-linked immunosorbent assay

Viral Symptoms	SARS-CoV-2 ELISA result		
	Reactive at Baseline	Reactive at 60 days	Mean change in percentage from seropositive to seronegative
Symptomatic	35 (48.6%)	20 (54.1%)	- 42.8%
Asymptomatic	37 (51.4%)	17 (45.9%)	- 54.0%

## Discussion

SARS-CoV-2, a coronavirus, presents with signs and symptoms depending on the organ system involved. Respiratory system symptoms, high-grade fever, sore throat, nonproductive cough, shortness of breathing, diarrhea, and fatigue are often initial presenting complaints with neurological involvement or patients being asymptomatic also common. Air droplets or direct contact remains the common mode of transmission [9]. With a mean incubation period of 5.1 days, 97.5 percent of the patients develop symptoms from SARS-CoV-2 infection within 11.5 days, implying​ that a quarantine period of 14 days is required in suspected cases to potentially expose the infection [10]. Nucleic​ acid amplification diagnostic testing or real-time PCR of samples collected from suspected patient’s nasal and pharyngeal swabs or sputum remains the gold standard for diagnosis as recommended by World Health Organization (WHO) [11]. Serologic studies using ELISA are often also used as a screening test for SARS-CoV-2 infection, which however have not been proven to be solely used to diagnose or exclude the infection or to inform infection status [4,5].

The present study was conducted to see whether antibodies against COVID-19 or its serology are protective in the long term or not. It concluded that out of 72 participants who were included in the study and were seropositive at baseline, 37 (51.3%) remained seropositive after 60 days with almost equally (48.6%) becoming seronegative, which indicates that there might be a chance of no long-term protection from SARS-CoV-2 infection. Sero-negativity after 60 days was more commonly (54% vs 42.8%) seen in individuals who were asymptomatic as compared to individuals who were symptomatic at presentation. In their study to assess the duration of antibody response to SARS-CoV-2 infection in health care personnel, Patel et al. concluded that eight (42%) out of 19 seropositive individuals at baseline remained seropositive after 60 days while 11 (58%) became seronegative. Of these 11 who became seronegative, six (55%) were asymptomatic at presentation while only two (25%) were asymptomatic in individuals who remained seropositive, indicating high seroconversion in asymptomatic individuals [12].

Antibody decay was found to be quicker after SARS-CoV-2 infection than that reported for SARS-CoV-1 [13,14]. Long et al. in their study to assess the immunological response of​ asymptomatic individuals infected with SARS-CoV-2 concluded that asymptomatic ​individuals had a weaker immune response due to long duration of viral shedding, significantly lower virus-specific IgG levels in the acute phase, and greater reduction in the IgG and neutralizing antibodies during the early convalescent phase of which 40% became seronegative during the same phase [14]. 

These findings indicate that humoral immunity against SARS-CoV-2 may not be long-lasting among individuals with mild illness, who compose the majority of cases with COVID-19 [13]. Also, serology tests cannot be solely used to diagnose or exclude the infection or to inform infection status. 

## Conclusions

In this study, almost half of the patients became seronegative for SARS-CoV-2 after two months. Mean change in percentage from seropositive to seronegative was greater in asymptomatic compared to symptomatic patients. Our study suggests that presence of immunoglobulins in the serum do not provide long-lasting immunity and there might be a chance of reinfection. Based on this result, we suggests patients who have recovered from COVID-19 should continue precautionary measures. 
